# Telerehabilitation for upper limb disabilities: a scoping review on functions, outcomes, and evaluation methods

**DOI:** 10.1186/s13690-022-00952-w

**Published:** 2022-08-23

**Authors:** Khadijeh Moulaei, Abbas Sheikhtaheri, Mansour Shahabi Nezhad, AliAkbar Haghdoost, Mohammad Gheysari, Kambiz Bahaadinbeigy

**Affiliations:** 1 Medical Informatics Research Center, Institute for Futures Studies in Health, Kerman University of Medical Sciences, Kerman, Iran; 2 Department of Health Information Management, School of Health Management and Information Sciences, Iran University of Medical Sciences, Tehran, Iran; 3 Department of Physical Therapy, Faculty of Allied Medicine, Kerman University of Medical Sciences, Kerman, Iran; 4 HIV/STI Surveillance Research Center and WHO Collaborating Center for HIV Surveillance, Institute for Futures Studies in Health, Kerman University of Medical Sciences, Kerman, Iran; 5 Business Administration Management (Digital Transformation), Faculty of Management, Tehran University, Tehran, Iran; 6 Medical Informatics Research Center, Institute for Futures Studies in Health, Kerman University of Medical Sciences, Kerman, Iran

**Keywords:** Telerehabilitation, Rehabilitation, Upper limb, Disabilities, Telemedicine, Digital health

## Abstract

**Background:**

Upper limb (UL) disabilities have attracted worldwide attention due to the high economic costs of health care and the negative effects on the quality of life of patients with these disabilities. Telerehabilitation technologies are one of the most important ways to reduce rehabilitation costs and increase the quality of life of patients. Therefore, the aim of this study was to investigate the role of telerehabilitation in improving the health status of patients with upper limb disabilities.

**Methods:**

This scoping review was conducted by searching the Web of Science, PubMed, and Scopus until July 30, 2021. We used a data extraction form with 18 fields to extract data from primary studies. The selection of articles and data extraction was made by four researchers using a data collection form based on inclusion and exclusion criteria. Disagreements were resolved through consultation with the fifth and sixth researchers.Inclusion criteria were studies published in English, studies on upper limb disability, and telerehabilitation based on any technology (synchronous telerehabilitation, asynchronous, or both). Exclusion criteria were articles that did not focus on telerehabilitation and upper limb disabilities. Also, books, book chapters, letters to the editor, and conference abstracts were also removed.

**Results:**

A total of 458 articles were retrieved, and after removing irrelevant and duplicate articles, 29 articles were finally included in this review. Most telerehabilitation was performed for patients with stroke (65%). Among the 15 different services provided with telerehabilitation technologies, "Evaluation of exercises and also a musculoskeletal function of patients by the therapist","Recording of patients' rehabilitation exercises and sending them to the therapist” and "Prescribing new rehabilitation exercises by the therapist" were the most widely used services, respectively. Virtual reality technologies, smart wearables, and robots were used to provide telerehabilitation services. Among the 13 types of evaluation used for telerehabilitation systems, “Evaluation and measurement of upper limb function” was the most used evaluation in the studies. "Improvement in musculoskeletal functions”, "Increasing patients' interest and motivation to perform rehabilitation exercises", and "Increasing adherence to rehabilitation exercises and greater participation in treatment processes" were the most important outcomes, respectively.

**Conclusion:**

Our findings indicate that telerehabilitation provides individuals with equitable access to rehabilitation services, improves musculoskeletal function, and empowers individuals by providing a variety of rehabilitation capabilities.

**Supplementary Information:**

The online version contains supplementary material available at 10.1186/s13690-022-00952-w.

## Background

Upper limb (UL) disability is a common health problem in the general population [1]. Upper limb disabilities have attracted worldwide attention due to the high economic costs of their health care and negative effects on the individuals’ quality of life [1]. Patients with these disabilities, on the other hand, have a shorter period of illness and are discharged from hospitals sooner than in the past. This is mainly due to the time limits and economic considerations that health organizations are facing. However, many of these patients still need rehabilitation services to fully recover from the disease. In addition, such services are often costly and patients sometimes have to make several travels to a rehabilitation center during the treatment process [2].

Technology advancements have enabled new approaches to rehabilitate upper limb disabilities. Internet access and video conferencing have grown in popularity over the last decade, allowing for remote training for people with upper limb disabilities [3]. Telerehabilitation, as an emerging field of telemedicine, is defined as a set of tools, procedures, and protocols for providing rehabilitative processes remotely [4]. Telerehabilitation has the potential to provide rehabilitation services that go beyond conventional treatment and address the current functional needs of patients with chronic upper extremity injuries [3]. The technology improves service quality by monitoring patients on-site, mainly in communities far from urban centers. This technology is also expected to improve the cost-effectiveness of interventions [5]. Previous systematic studies evaluated the feasibility, effectiveness, and cost issues of telerehabilitation for individuals with a variety of health conditions. The results of these studies showed that remote rehabilitation is an effective alternative to face-to-face interventions [6, 7].

To our knowledge, no comprehensive review has been published on the telerehabilitation of upper limb disabilities as yet. As a result, the purpose of this scoping review is to identify the functions and outcomes of telerehabilitation intervention in the recovery of upper limb functions. In addition, we identified various evaluation methods for telerehabilitation systems in this study.

## Material and methods

The current study is a scoping review on the functions and outcomes of telerehabilitation in the improvement of upper limb disabilities. Scoping reviews are an effective and useful method for determining the scope or coverage of a body of literature on a specific topic, as well as providing a clear indication of the volume of literature and studies available, and an overview (broad or detailed) of its focus [8]. It should be noted that we used the PRISMA scoping reviews checklist for selecting studies and reporting the results [9].

### Information sources and search strategy

To find articles about telerehabilitation for upper limb disabilities, three databases were searched: PubMed, Web of Science, and Scopus until January 3, 2022. The keywords upper limb disability and telerehabilitation were used to conduct the search process. The search strategy was developed by three researchers (KB, AH, and ASH) and finally approved by KHM and ASH. The keywords and search strategies used in each database are listed in Additional file 1: Appendix A. In January 2022, the search was carried out across all three databases. Furthermore, we did not impose any restrictions on the database search's starting point. It should be noted that in order to access articles that we did not have full-text access to, we emailed the corresponding author and requested that they send them to us.

### Eligibility criteria

In this study, articles focusing on telerehabilitation and upper limb disability were included. Inclusion criteria included articles published in English, research on upper limb disability due to any disease or injury, telerehabilitation based on any technology (robotics, smart wearables, and virtual reality), and synchronous telerehabilitation (real-time or live video) and asynchronous (stored-and-forward). Exclusion criteria also included articles that did not focus on telerehabilitation and upper limb disabilities. Books, book chapters, letters to the editor, and conference abstracts were also removed.

### Selection of sources of evidence

First, abstracts of all retrieved articles were entered into EndNote software and duplicate articles were excluded by KHM. Then, according to the inclusion and exclusion criteria, title and abstract of the articles, related studies were selected by AH, MGH, and MSH. All valid articles included in the study were reviewed by KB and KHM (authors reviewed an article together) and finally approved by ASH. In case of disagreement, the final decision on each article would be decided by discussion between the authors. After the final approval of the articles, their full texts were reviewed by KHM and ASH to extract the required data.

### Data charting process and data items

We used a data extraction form to extract data from primary studies. The validity of this form was confirmed by two medical informatics and health information management specialists. Data extraction form includes fields such as country, publication year, the purpose of study, disease leading to upper limb disability, upper limb part involved in the disability (Table 1 and more details in Additional file 1: appendix B), functions and services of telerehabilitation, services provided with telerehabilitation systems (Table 2 and more details in Additional file 1: appendix B), hardware equipment used in providing telerehabilitation services (Additional file 1: appendix B), assistive technologies to provide telerehabilitation services, types of services (synchronous and asynchronous, synchronous or asynchronous) (Table 3 and more details in Additional file 1: appendix B), equipment used to build the robot, use of smart wearables devices type of smart wearable devices, equipment used to make the smart wearable devices (Additional file 1: appendix B), duration of use of the telerehabilitation systems (Additional file 1: appendix C), evaluation and type of evaluation, samples size ( Table 4 and more details in Additional file 1: appendix C), outcomes and results ( Table 5 and more details in Additional file 1: appendix C).

### Data collation process

After the final approval of the articles in the previous steps, in order to extract the required information, their full text was read separately by KB, KHM, and ASH. Necessary information was extracted from the articles and recorded in the above-mentioned data extraction form (KB, KHM and ASH). Then, the information extracted from the articles was re-examined separately by MGH and MSH and confirmed by AH. When there was disagreement about the information extracted, the members of the research team met to make a final decision. It should be noted that for articles with missing information, we emailed the corresponding author and asked them to send us the missing information. Finally, the extracted information was entered into an Excel spreadsheet.

### Synthesis of results

After entering the extracted data into an Excel file, we qualitatively classified the various telerehabilitation applications in patients with upper limb disabilities and reported their results and frequency. To synthesize data, the authors used the advancement scoping method recommended by Levac et al. [10]. One author (KHM) refined the data (e.g., spell check, cell formatting) to ensure that Excel performed procedures, calculations, and analyses correctly and adequately (e.g., making axis tables, and charts). Because scoping reviews do not seek to summarize or weigh evidence from various studies [10, 11], only descriptive analyses (e.g., frequencies, percentages) were performed on the extracted data to describe the findings of the included studies [12]. The descriptive data and findings from the included articles were organized into tables and figures based on themes to provide the review's findings, which guided the study objectives (by KB, KHM, and ASH). If the authors disagreed, the final decision on each figure or table was reached through discussion.

### Ethical considerations

The protocol of this study was approved by the ethical committee of Kerman University of Medical Sciences (IR.KMU.REC.1400.606).

## Results

### Selection of sources of evidence

**Fig. 1:**
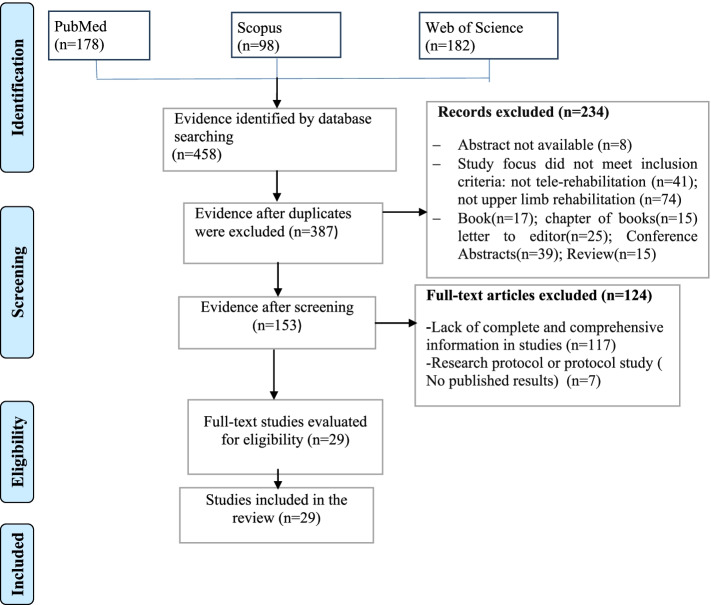
Study selection process

In total, 458 articles were retrieved. After removing duplicates, the remaining 387 studies were carefully reviewed and assessed based on inclusion and exclusion criteria. Finally, 29 articles were included in the study. The results of the search and study selection are presented in Fig. 1.

### Characteristics of the included studies

An overview of selected studies is presented in Table 1.

**Table 1:** Overview of studies included in the study

Author(s)	Country	Year	Study aim	Disease leading upper limb disability	Upper limb part involved in the disability
Piron [13]	USA	2004	To assess the impacts of a telerehabilitation technology for the treatment of arm motor disabilities due to a stroke	Stroke	Arm
Reinkensmeyer [14]	USA	2002	To establish a web-based telerehabilitation for upper limb disabilities after stroke	Stroke	Arm and hand
Feng [15]	USA	2005	To design and development of UniTherapy as a telerehabilitation system	Stroke	UL
Kuttuva [16]	USA	2006	To design and development of the Rutgers Arm as a training system for shoulder rehabilitation exercises	Stroke	UL
Pickett [17]	Columbia	2007	To examining the impact of clinic-based Constraint-Induced Movement Therapy (CIMT) and a home-based CIMT trial (teleCIMT) consolidating telecommunications innovation in progressing the performance of two stroke survivors	Stroke	UL
Page [18]	US	2007	To determining the effectiveness of modified constraint-induced therapy extension (mCITE) protocol in telerehabilitation of patients with stroke	Stroke	Arm
Sucar [19]	Mexico	2009	To design and development of a low-cost telerehabilitation system for arm disability rehabilitation in stroke survivors that allows them to perform arm physical exercise at home or in the medical centers with cyclic interactions with the therapist	Stroke	Arm
Sucar [20]	Mexico	2010	To helping people with a stroke to do arm exercising at home or clinical centers without an advisor	Stroke	Arm
Golomb [21]	USA	2010	To explore whether in-home remotely checked virtual reality videogame-based telerehabilitation in youths with spastic hemiplegia can progress hand work and lower arm bone wellbeing, and illustrate changes in motor circuitry stimulation	Stroke(Hemiplegic Cerebral Palsy)	Forearm and hand
Golomb [22]	USA	2011	The evaluation of hand performance and arm bone wellness after 14 months of intervention through video game-based telerehabilitation in teenagers with spastic hemiplegia	Stroke(Hemiplegic Cerebral Palsy)	Hand
Aung [23]	Australia	2012	To design and evalution of a successful augmented reality based upper extremity's rehabilitation framework with low cost	Traumatic Brain Injury (TBI), Spinal Cord Injury (SCI) and Cerebrovascular Accident (CVA) or stroke	UL
Sampson [24]	New Zealand	2012	To design and improvement of upper extremities rehabilitation using bilateral exercise and the Bilateral Upper Limb Coach (BUiLT) to supply symmetrical and two-sided arm training in a "constrained" and self-assisted way	Stroke with hemiparesis	UL
Langan [3]	USA	2013	To examines the employ of telerehabilitation to move forward upper extremities function in unremitting periods of stroke recuperation	Stroke	UL
Piga [25]	Italia	2014	To self-managed kinesiotherapy meetings for the recovery of hand performance in patients with systemic sclerosis (SSc) and rheumatic joint pain sensation	Systemic Sclerosis and Rheumatoid Arthritis	Hand
Langan [26]	USA	2014	To evaluation of upper extremity performance in adults with hemiplegic cerebral dysfunction after home-based telerehabilitation	Cerebral Palsy	Arm
Tousignant [27]	Canada	2014	To feasibility assessment a home tele-treatment program for people with humeral fractures	Proximal humerus fractures	Arm
Kato [28]	Japan	2015	To establishment of a telerehabilitation method based on virtual reality technology for rehabilitation of paralysed upper and lower limbs and poor balance in people with stroke	Stroke	UL
Van Straaten [29]	US	2015	To testing the powerfulness of the high-dose practice program provided through telerehabilitation for patients with SCI and defining whether the intervention reduces pain and increases performance	SCI	Arm
Cabana [30]	Canada	2016	To evaluation of clinical effects of telerehabilitation approach (TELE group) in comparison with face-to-face visits to a clinic for patients undergoing proximal humerus fracture	Proximal humerus fracture	Arm
Tsekleves [31]	UK	2016	The development and assessment of a low-cost VR-based telerehabilitation platform for stroke patients with upper extremity disabilities	Stroke	UL
Song [32]	China	2016	To design of a new one-therapist to three-patient robot for remote rehabilitation of patients with upper limb disability	Stroke	UL
Lambert [33]	Australia	2017	To measurement of adherence to home practice programmes for sick person with musculoskeletal disorders with telerehabilitation program compared to paper handouts	Musculoskeletal	UL
Kizony [34]	Israel	2017	To evaluation of the effect of telerehabilitation services in patients with Acquired Brain Injury (ABI) with upper limb disabilities for two months or more	Acquired Brain Injury (ABI)	UL
Cikajlo [35]	Slovenia	2018	To exploring the role of telerehabilitation games in functional enhancement of upper limb disabilities in people with Parkinson’s diseas	Parkinson’s disease (PD)	UL
Kim [36]	South Korea	2018	To evaluate the effectiveness of the Kinect-based virtual rehabilitation (VR) system for upper extremities healing in patients with acute stroke	Subacute stroke	UL
Cabrera-Martos [37]	USA	2019	To assess the degree of compatibility between face-to-face and telerehabilitation-evaluation of UL performance in patients with PD	Parkinson disease (PD)	Hand or fingers; and/or previous trauma or fracture of the upper extremities
Hung [38]	Taiwan	2019	To create Kinect2Scratch games and compare the impacts of training with a therapist-based training on the upper extremity performance of patients with stroke	Stroke(Hemiplegic Cerebral Palsy)	Shoulders, elbows and wrists
Agyeman [39]	UK	2019	To design and development of a wearable device for propelling patients with upper and/or lower limb Inability through gaming and home-based telerehabilitation	Stroke	UL
Qiu [40]	US	2020	To design and create of the home based virtual rehabilitation system (HoVRS) to telerehabilitation and custom-made upper extremity training	Stroke	Elbows, shoulders, wrists, hands and arms

**Fig. 2:**
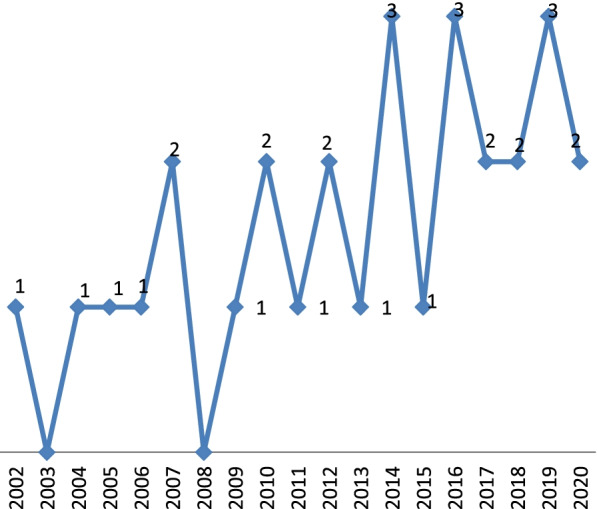
Distribution of the studies in terms of publication year

As shown in Fig. 2, telerehabilitation technologies are used to rehabilitate upper limb disabilities since 2002. Most articles were published in 2014 [25–27], 2016 [30–32] and 2019 [37–39](*n* = 3). No studies were published in 2003 or 2008. (More details in Table 1).

The majority of studies were conducted in the United States (*n* = 12, 41%) [3, 13–16, 18, 21, 22, 26, 29, 37, 40]. Also, 6.88% of studies were carried out in Australia (*n* = 2) [23, 33]. South Korea, China, Taiwan, and Japan are the only Asian countries to conduct telerehabilitation studies for upper limb disabilities limb (see Table 1 for more details).

**Fig. 3:**
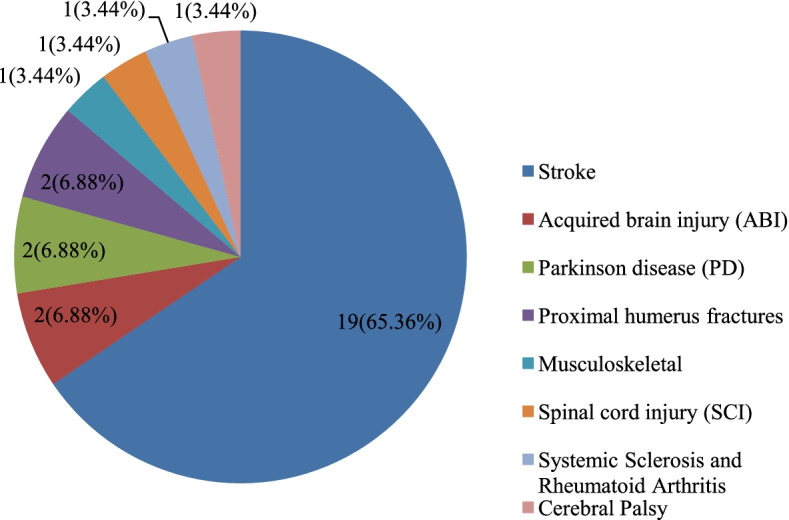
The distribution of the studies based on diseases and injuries leading to upper limb disabilities

As shown in Fig. 3, telerehabilitation was performed for eight categories of diseases or complications leading to upper limb disabilities. Most telerehabilitation was performed for patients with stroke (*n* = 19 (65%) [3, 13–22, 24, 28, 31, 32, 36, 38–40]. The frequencies and percentages of other diseases are shown in Fig. 3.

### Functions and services of telerehabilitation

A total of 15 different functions and services were identified for telerehabilitation systems. "Evaluation of exercises and also a musculoskeletal function of the patient by the therapist” (*n* = 19), "Recording of patients' rehabilitation exercises and sending them to the therapist" (*n* = 16), and "Prescribing new rehabilitation exercises by the therapist "(*n* = 16) were the most widely used functions and services of telerehabilitation systems for rehabilitation of upper limb disabilities, respectively. (Table 2, more details in Additional file 1: Appendix B).

**Table 2:** Overview of the functions and services of telerehabilitation presented in the studies

Functions and services of telerehabilitation	References	Frequency of telerehabilitation functions based on the number of references
Evaluation of exercises and musculoskeletal function of the patient by the therapist synchronously and asynchronously (Retrieval of the patient's recorded treatment exercises by the therapist)	[13, 15–24, 26, 31, 32, 35–37, 39, 40]	19
Recording of patients' rehabilitation exercises and sending them to the therapist	[13–16, 18, 19, 22, 24–26, 30–33, 39, 40]	16
Prescribing new rehabilitation exercises by the therapist	[14, 15, 18–22, 24, 26, 29, 31–33, 36, 39, 40]	16
Real-time face-to-face communication between patients and therapists	[13, 15, 17, 23, 27–30, 32, 37, 40]	11
Providing the educational information to patients in text, video and audio formats to do rehabilitation exercises	[3, 14, 21, 25, 32, 34, 38]	7
Providing the progress charts to the patient and therapist	[14, 18–20, 40]	5
Providing the motivational text and voice messages (such as encouragement or applause) to encourage the patient to perform rehabilitation exercises	[14, 31, 33, 34, 38]	5
Set rehabilitation games and exercises by the therapist based on the patient's ability and performance	[34–36]	3
Ability to select rehabilitation exercises or games by the patient based on the level of disability or injury	[31, 36, 39]	3
Determine range of motion, speed, duration, number of repetitions, scoring and feedback for each rehabilitation exercise	[25, 31]	2
Patient access to history of rehabilitation exercises	[32]	1
Automatic alert to patients to perform compensatory movements	[38]	1
Show the number of correct movements to the patient	[38]	1
3D display of images of the UL	[31]	1
Recording and reporting of rehabilitation exercises performed by the patient	[40]	1

### Methods of providing telerehabilitation services and exercises

According to Table 3, virtual reality technologies, smart wearable devices, and robots were used to provide telerehabilitation services. Virtual reality was the most widely used technology. Different types of rehabilitation services were provided to patients in three forms: synchronous, asynchronous, and a combination of synchronous and asynchronous. Combined synchronous and asynchronous services were the most common type of services. (More details in Additional file 1: Appendix B).

**Table 3:** Assistive technologies to provide telerehabilitation services and types of telerehabilitation services

Assistive technologies to provide telerehabilitation services and type of telerehabilitation services		References	Frequency of assistive technologies and type of telerehabilitation service based on the number of references
**Assistive technologies to provide telerehabilitation services**	**Virtual reality**	[3, 15, 16, 19–24, 28, 31–36, 38–40]	19
	**Smart wearables**	[13, 21, 22, 28, 39]	5
	**Robots**	[32]	1
**Types of telerehabilitation service**	Synchronous and asynchronous	[15, 16, 19–22, 26, 31, 33, 36, 38–40]	13
	Synchronous	[3, 13, 17, 23, 24, 27–30, 32, 33, 37]	12
	Asynchronous	[14, 18, 19, 25, 31, 34, 35]	7

### Evaluations in telerehabilitation systems for upper limb disabilities

The number of participants in the evaluation of telerehabilitation systems varied from one to 85 people. Also, the minimum and maximum duration of the evaluation processes were 20 min [37] to 14 months [22], respectively. According to Table 4, thirteen different types of evaluations were performed for telerehabilitation systems in upper limb rehabilitation. “Evaluation and measurement of upper limb function” was the most common type of evaluation (*n* = 21). “Fugl-Meyer Upper Extremity score (FMA-UE)” was the most widely used evaluation method in the measurement of upper limb function (*n* = 9). Evaluation of “Range and motor skills and functional strength of the hand” was in the next rank (*n* = 7). As shown in Table 4, 8 different evaluation methods were used. After "Range and motor skills and functional strength of the hand", “Lab-based clinical and kinematic “ and “Patient satisfaction” were the most used evaluation methods (*n* = 5). Questionnaires and interviews were used to assess patient satisfaction. Questionnaire (*n* = 3) was used more than interview (*n* = 1). (More details in Additional file 1: Appendix C).

**Table 4:** Types of evaluation in telerehabilitation systems for rehabilitation of upper limb disabilities

Evaluation types	Evaluation Methods/tools	References	Frequency of evaluation tools / methods based on the number of references	All References for evaluation types	Total frequency of types of evaluation based on the number of references
**Evaluation and measurement of upper limb function**	Fugl-Meyer Upper Extremity score (FMA-UE)	[13, 16, 17, 19, 24, 29, 34, 36, 38]	9	[3, 13, 15–25, 27, 29, 30, 34–38]	21
	Wolf Motor Function (WMFT)	[3, 18, 19, 38]	4		
	Disabilities of Arm, Shoulder, and Hand (DASH)	[27, 29, 30]	3		
	Motor Activity Log (MAL)	[17, 18, 38]	3		
	Finger range of motion (ROM)	[21, 30]	2		
	Use of EMG sensors and other sensors	[21, 23]	2		
	Box and Block Test (BBT)	[17, 35]	2		
	Parkinson’s Disease Questionnaire (PDQ-39)	[35]	1		
	Wheelchair User’s Shoulder Pain Index (WUSPI)	[29]	1		
	Intrinsic Motivation Inventory (IMI)	[24]	1		
	Index, and Shoulder Rating Questionnaire (SRQ)	[29]	1		
	Unified Parkinson’s Disease Rating Scale (UPDRS)	[35]	1		
	Stroke Impact Scale (SIS)	[17]	1		
	Hand Mobility in Scleroderma (HAMIS)	[25]	1		
	Functional Index of Hand Osteoarthritis (FIHOA)	[25]	1		
	Jebsen-Taylor Hand Function Test (JTHFT)	[22, 35]	2		
	Actual Amount of Use Test (AAUT)	[17]	1		
	Nine-Hole Peg Hole Test (9HPT)	[35]	1		
	Motricity Index (MI)	[20]	1		
	Manual ability measure, dexterity (evaluated using the coin rotation task), motor speed (assessed by the finger tapping test), tremor (evaluated with the Fahn-Tolosa-Marin Tremor Rating Scale), and range of motion (using the Kinovea software(	[37]	1		
**Range and motor skills and functional strength of the hand**	Unilateral reaching movements from a waist to shoulder height target using an electro-magnetic 3D motion monitor (Ascension Technology Corporation, Burlington, VT) [3]	4]	1	[3, 13, 17, 19, 21, 32, 34]	7
	Determination of the velocity and duration of 10 representative reaching movements(simple movements, e.g. pouring water from a glass, using a hammer, turning around the centre of a doughnut, etc.)	[13]	1		
	Measuring arm movement and interactive force between the patient arm and the robot	[32]	1		
	Grip strength assessment using a standard grip dynamometer	[17]	1		
	Bruininks-Oseretsky Test of Motor Proficiency	[21]	1		
	Trail Making Test (TMT, parts A and B)	[34]	1		
	Point quality of movement (QOM)	[19]	1		
	By glove sensors	[21]	1		
**Lab-based clinical and Kinematic Measures**	Dual energy x-ray absorptiometry (DXA)	[21, 22]	2	[3, 21, 22]	4
	Laboratory-based pre-, post- and one-month follow-up testing	[3]	1		
	Peripheral quantitative computed tomography (pQCT)	[21]	1		
	Functional magnetic resonance imaging (fMRI)	[21]	1		
	Computed tomography(CT)	[22]	1		
**Patient satisfaction**	Questionnaire	[27, 30]	2	[18, 27, 30, 33]	4
	Health Care Satisfaction Questionnaire				
	Non-standard tools	[33]	1		
	Interview	[18]	1		
	Structured interview				
**Perceived treatment effectiveness, and different aspects of adherence**	Semi-structured interview	[19, 33]	2	[19, 33]	2
	Online survey	[33]	1		
**Limb position sense assessment**	By moving different parts of the upper limb	[26]	1	[26]	1
	One arm was passively extended 30° (reference position), held for three seconds and then returned to the original position	[3]	1	[3]	1
**Standardized occupational therapy assessments**	Using a Sammons Preston Jamar dynamometer and pinchometer	[21]	1	[21]	1
**Severity of pain**	Short-Form McGill Pain Questionnaire [SF-MPQ])	[27]	1	[27]	1
**Usability testing**	System Usability Scale (SUS) Questioner	[34]	1	[34]	1
**Accuracy and precision of the system**	Pilot test	[31]	1	[31]	1
**Feasibility of the use of system**	Pilot test	[40]	1	[40]	1
**Cost analysis**	Cost of services(Questionnaire)	[30]	1	[30]	1
**Quality of life**	Stroke Impact Scale (SIS), Evaluating the impact of the programs on quality of life after the intervention and a follow-up	[17]	1	[17]	1

### Outcomes of using telerehabilitation systems for upper limb disabilities

The most important outcomes of using telerehabilitation systems were, in order, "improvement in musculoskeletal functions," “increasing patients' interest and motivation to perform rehabilitation exercises", and "increasing adherence to rehabilitation exercises and greater participation in treatment processes." Table 5 shows the other outcomes. (More information can be found in Additional file 1: Appendix C.)

**Table 5:** Outcomes of using telerehabilitation systems

Outcomes	References	Outcomes frequency based on the number of references
Improvement in musculoskeletal functions (Musculoskeletal strength, sensation, perception, flexibility and range of motion)	[3, 13, 15–17, 19–26, 28, 31–38, 40]	23
Increasing patients' interest and motivation to perform rehabilitation exercises	[3, 16, 19, 20, 23, 24, 31, 34, 38, 40]	10
Increasing adherence to rehabilitation exercises and more participation in treatment processes	[17, 18, 25, 29, 31, 33, 40]	7
Improved user satisfaction	[3, 13, 18, 27, 33, 34]	6
No adverse effect on patients	[28, 31, 38, 40]	4
Telerehabilitation systems feasibility for remote monitoring and control of patients	[14, 18, 40]	3
Reduced or relieved pain	[19, 27, 29]	3
Reliability of telerehabilitation systems	[24, 32, 37]	3
Improved quality of life	[17, 35]	2

It should be noted that “Increasing the total exercise time" was the only negative outcome reported in one of the 29 studies included in our study [16].

## Discussion

The functions and outcomes of telerehabilitation technology for the upper limbs were investigated in this study. In addition, in this study, we identified different methods for evaluating telerehabilitation. "Evaluation of exercises and musculoskeletal function of the patient by the therapist" was the most widely used function/service provided by telerehabilitation. Three technologies of virtual reality, smart wearables, and robots were used to provide telerehabilitation services. Among the 13 different evaluations of telerehabilitation systems, "Evaluation and measurement of upper limb function" was the most widely used. Researchers reported a variety of outcomes of telerehabilitation systems for upper limb disabilities. These outcomes are described below.

- ***Improvement in musculoskeletal functions***

One of the most important findings of the studies reviewed in this study was improving musculoskeletal functions through telerehabilitation. When compared to usual and traditional care, these findings highlight the potential for telerehabilitation to be an alternative model of care. As a result, while telerehabilitation may not be superior to traditional models of care, it may provide additional benefits in clinical settings (such as increased access to health care for patients and improved efficiency for health professionals). However, to increase the effectiveness of telerehabilitation in improving musculoskeletal functions, a person-centered approach to treatment should be employed to encourage greater participation in exercise [41]. Different studies [42, 43] suggest that patients prefer individualized, supervised exercise programs with a therapist's opinion. In addition to web-based software, the development of mobile-based telerehabilitation applications can provide patients with personalized and supervised exercise programs and input from therapists [44]. There is evidence that using mobile apps with therapist input, particularly the ability to set and monitor the quality of exercise completion [33], leads to higher adherence rates and thus improves musculoskeletal functions than traditional paper handouts. This success could be attributed to a variety of factors, including the ability of applications to send alerts, motivating messages, or reminders. Furthermore, patients may prefer to access their home exercise programs (HEPs) via a mobile phone or another portable device rather than a paper handout [33]. Patients' adherence, on the other hand, maybe positively affected if they are aware that their therapist can remotely monitor and provide feedback via an application [45].

In a systematic review, Sarfo et al., [46] concluded that telerehabilitation interventions significantly improved motor deficits, higher cortical dysfunction, and people with upper limb. However, Some studies have reported that telerehabilitation systems may not improve upper limb mobility and function in the short term, and the long-term effectiveness of these systems may be apparent to patients [19]. Long-term maintenance of motor gains or improvement in musculoskeletal functions includes continued adherence to exercises (after the end of telerehabilitation) and maintaining motivation in the absence of external factors (e.g., clinician encouragement) [47]. Musculoskeletal functions and upper limb range of motion can be improved in these systems by incorporating games and virtual reality technology [38]. By controlling movement, increasing the number of repetitions of exercises, and motivating patients, video games can improve musculoskeletal function and range of motion [38]. According to these studies, it can be said that the therapeutic effects of these systems are not less than traditional systems, but the effectiveness of these telerehabilitation systems may not be apparent in the short term. On the other hand, with the use of video games based on virtual reality technologies and mobile-based applications, the therapeutic effects of these systems will increase.

- ***Increased patients’ interest and motivation to perform rehabilitation exercises***

Other findings of our study showed that telerehabilitation will increase patients' interest and motivation to perform therapeutic exercises. Aguilera-Rubio et al. [48] demonstrated that motivation has an effect on motor and functional outcomes in people with orthopedic and neurological disorders. Some of studies [49] show that a lack of motivation is a barrier to physical activity and training following a disability. Sucar et al. [19], also reviewed the Gesture Therapy (GT) telerehabilitation system versus traditional rehabilitation. They concluded that there was a greater motivation and dependence on treatment for patients using GT. They also noted that motivation and long-term rehabilitation exercises are key factors in improving upper limb function. Low levels of motivation have a negative effect on the type and extent of use of rehabilitation exercises [19]. Therefore, motivation factors should be considered in rehabilitation services [40]. Video game exercises are one of the most important reasons for strengthening and motivating participants to do rehabilitation exercises [38, 40]. Visual feedback provided through games increases the patients’ awareness of their progress and increases their motivation and their continuity in treatment. In addition, visual biofeedback (for example, tools like Kinect) promotes higher levels of active participation [38]. Patients who use virtual environment video games are more motivated, rely more on rehabilitative exercises [50, 51], enjoy treatment more, work harder to heal themselves, and ultimately have better upper limb function [19, 52]*.* Some studies [48, 53] have also shown that VR-based games can provide effective sensory feedback and place subjects in a virtual environment to watch their body’s movements. On the other hand, the virtual and gaming environment should be designed in such a way that the patient has a sense of presence in a real environment. Considering other elements such as the background, avatar design, and the scene’s realism are very important in designing virtual reality and game environments [47]. Personalization of rehabilitation programs and games by actively involving the patient in setting rehabilitation goals may thus directly increase the patient’s sense of independence and in turn, their motivation, as suggested by Jansson et al. [54].

- ***Increased adherence to rehabilitation exercises and greater participation in treatment processes***

Of the 29 studies, seven studies showed that telerehabilitation increased adherence to rehabilitation exercises and increased patient participation in their treatment processes. The results of the study by Lambert et al. [33] showed that participants who received their rehabilitation exercises through telerehabilitation were more adherent to treatment and their performances were improved more than participants who received paper handouts. Cramer et al., [55] also examined the effectiveness of home-based telerehabilitation versus traditional in-clinic for adults with stroke. The patients in the telerehabilitation group had 35.4 (98.3%) of the 36 assigned treatments, and the patients in the clinical group had 33.6 (93.3%) of the 36 assigned treatments. At the same time, poor adherence to home exercise programs and treatment plans may affect treatment outcomes and symptom recurrence [56]. Non-adherence to a home exercise program may be due to patient-related reasons, including pain, lack of motivation, poor self-efficacy, limited exercise experience, and reduced social support. Furthermore, patients may not immediately recognize the benefits of a home exercise program [33]. Some authors [57, 58] have suggested that adherence to a home exercise program could be enhanced if therapists increase face-to-face time with patients, but this is costly and rarely feasible given limited resources. Therefore, the other effective factors in increasing adherence should be better understood and strategies should be provided to stimulate long-run exercises. Palazzo et al., [56] presented the physical exercise program (number, power, complexity, and weight of exercising), health care trip (breakdown between monitored meetings and home practice, lack of follow-up, and problems contacting care providers), diseased person representations (unwellness and representation of exercise, discouragement, natural depression, and lack of motivation), and the environment (attitudes of others, exercise planning problem) as the most important barriers to adherence to rehabilitation exercises that must be considered when designing and implementing telerehabilitation systems. Apart from environmental and exercise-related factors, various aspects of technology will also affect adherence to treatment and rehabilitation exercises. Qiu et al. [40] believe that adherence to rehabilitation provided through technology has a complex structure because technical problems can be a major obstacle to increase adherence. Therefore, in designing technology, factors related to clinical-demographic characteristics of patients such as age, housing state, and level of computer expertise or degree of damage should be considered. In addition, an algorithm program operating in the background can dynamically change the difficulty level of games based on people's performance to limit frustration and increase their motivation [40]. Also, adherence to treatment processes can be increased if the system is easy for patients to use and has an attractive user interface [59]. Dodakian and et al. [59], believed that the best rehabilitation-treatment program is of little help to patients if they do not adhere to it, so telerehabilitation programs were designed to increase compliance. A key feature of this is the use of virtual reality gaming to drive therapy, a known way of maximizing desirability and accessibility and promoting patient engagement.

- ***Improved user satisfaction***

In this review, we observed that telerehabilitation can improve user satisfaction. In the study by Lambert et al. [33], approximately 90% of the people in the intervention group (who received their rehabilitation exercises through the website) expressed their satisfaction with healthcare service-support and healthcare service-delivery of more than 95%. In addition, a number of studies [60–62] found that telerehabilitation services are useful and effective both for children and adults with disabilities, and therapists have also reported high levels of satisfaction and acceptance of telerehabilitation services. Based on the findings of these studies and the study of Tousignant et al., [63] it can be said that satisfaction is an important indicator of the degree of efficiency and effectiveness, and its high level increases patient motivation and improves compliance with treatment. Satisfaction conceptualization defines both one's legitimate expectation of having demands met and one's perception of the actual experience [64]. Dislike some disagreements [65, 66], satisfaction is often used as one of the important factors in healthcare quality, as it can both influence adherence to treatment plans and improve clinical outcomes [67]. However, the concept of satisfaction is complex and is related to different aspects of healthcare such as accessibility of resources, qualification of healthcare professionals, the patient-therapist relationship, and the overall care organization [64].

Moreover, we believe that patient satisfaction with a health technology will not be easy; for high satisfaction, a number of factors must always be considered. Patients’trust in telerehabilitation, quality of the patient-therapist relationship, quality of rehabilitation sessions, quality and performance of the technological platform, and user-friendliness of equipment are among the factors that can affect patients’ satisfaction with telerehabilitation systems [63, 68]. Reducing disconnection problems, ease of use of telerehabilitation, absence of adverse events, adequacy of audio/video quality, and increased adherence to exercise training can also have a significant impact on improving patient satisfaction [69]. If the patient's satisfaction with a system such as telerehabilitation systems increases, its continuity and use increases, and the patient's' adherence to treatment processes and rehabilitation exercises will increase, resulting in the development of the system and its acceptance by users will be easy. Therefore, patient satisfaction with the designed systems should always be considered a basic principle of system development processes.

- ***No adverse effects on patients***

The studies examined also pointed out that telerehabilitation systems have no negative effect on patients. Qiu et al. [40] used the Virtual Rehabilitation System (HoVRS) to rehabilitate 15 patients with stroke. The results of this study showed that after 13.5 h of using the system, all 15 patients completed the rehabilitation process without any side effects. Results of a systematic review by Vieira et al. [70] showed that telerehabilitation improved functional capacity, performance, and physical factors of quality of life, and indeed significantly reduced side effects. In some other studies, Kinect2Scratch game-based training and therapist-based training were compared [38]. The results indicated that Kinect2Scratch game-based training did not cause any serious side effects for patients with upper extremity disabilities. Also, none of the patients needed further treatment [38]. Although in our study, studies reported no exercise-related adverse events in the telerehabilitation groups, other studies reported similar adverse events in the telerehabilitation system. Adverse events in a minority of participants included new-onset arrhythmias (including supraventricular contractions, atrial fibrillation, and premature ventricular contractions), hypertension, angina [71, 72], and worsening of chronic obstructive pulmonary disease (COPD) [73]. This may indicate that more monitoring is used at home than exercise. On the other hand, patients who experience side effects, in addition to their reluctance to use the technology, discontinue treatment or receive lower doses, both of which lead to an increase in the overall treatment dose [74]. Therefore, telerehabilitation systems should be designed in such a way that they can minimize side effects for patients and ensure the absence of side effects for patients before using the system so that they can use these systems safely.

- ***Telerehabilitation systems feasibility for remote monitoring and control of patients***

According to the findings of the studies, we found that the use of telerehabilitation systems for remote monitoring and control of patients with upper limb disabilities is feasible. Some studies [55, 59] have suggested that telerehabilitation for patients with stroke and upper limb disabilities are feasible and as effective as in-person therapy. Reinkensmeyer et al. [14] analyzed data from home-based use related to a stroke patient. The results of this analysis demonstrate that it is possible, useful, and effective to use the system to direct a medical care platform, mechanically assist movement, and track progress in movement power. This is while evidence demonstrates that the feasibility of telerehabilitation systems is not easy; Factors such as costs associated with purchasing, maintaining and insuring hardware, as well as logistics related to equipment provision, monitoring, and return, can affect the viability of a telerehabilitation system [75]. Computer training is another factor that can affect the feasibility of telerehabilitation systems [75]. Evidence indicates that computer training can significantly reduce computer anxiety while also increasing computer interest and performance in older adults [76]. This means that providing education and training to patients on relevant technologies can increase the use of telerehabilitation systems. In addition, access to computers at home and the age of individuals are both strongly related to individuals' feelings about the acceptance and feasibility of technology and telerehabilitation [75]. Access to a computer at home seems to be one of the strongest predictors of feasibility and acceptance. The study by Nelson et al. [75] showed that patients with access to computers were more interested in exercising learning, more confident in the technology, less likely to avoid technology, and more likely to engage and feel safe and secure in telerehabilitation system. Furthermore, this study showed that patients over the age of 65 were less likely to use a telerehabilitation system compared to patients under the age of 66 [75]. Their reluctance to use telerehabilitation systems is more likely because they are more worried, concerned, and less confident than their younger peers. As a result, in order for telerehabilitation systems to be feasible, this age group should not be excluded from consideration. To maximize the uptake of telerehabilitation systems, healthcare providers looking to implement telerehabilitation and the designers of these systems should consider patients' age, access to technology at home, training and education requirements, and technology preferences.

- ***Reduced or relieved pain***

In our review, some studies showed that telerehabilitation had an effect on reducing or relieving pain. The results of the study by Van Straaten et al [29] showed that after using the telerehabilitation system, the pain of patients decreased, the function of different parts of their upper limbs improved and the isometric power evaluation of the anterior serratus and scapular retractors increased. In the study by Tousignant et al. [27], the pain was assessed using the SF-MPQ test. The results of this study showed that the pain diminished essentially between face-to-face evaluations before (T1) and immediately after (T2) as demonstrated by the SF-MPQ score and the VAS. The illustrated pain reduction was more noteworthy than the negligible clinically significant contrast. In the study of Sucar et al. [19], the effect of using telerehabilitation and conventional occupational therapy systems in reducing pressure and pain was reported to be equal. In other studies [77, 78], aggressive rehabilitation programs have been shown to improve joint function and reduce pain, increase strength, walking speed, and self-efficacy, and reduce the risk of other chronic conditions. Both home-based exercise and aerobic walking reduce pain and disability [79].

But what is important is that we know that the pain does not reduce or alleviate itself by using telerehabilitation systems and that a number of indicators alongside these systems must always be considered. Osteras et al., [80] believed that high-repetition doses reduced pain more than lower doses. As a result, when considering the dose aspect of the dose-response relationship for telerehabilitation, we must consider items such as "number and duration of repetitions in one set," "number and duration of sets of an exercise," "number and duration of exercises in one treatment," "number and duration of treatments during a week (s)," and "total number and duration of treatments" [80]. While all of these indicators affect patients' treatment preferences, therapist contact mode proved a key driver of preference for chronic pain rehabilitation, with patients who have a high preference for face-to-face contact with some therapists. Treatment scenarios that include some remote therapist video communication are usually preferred over scenarios that only include remote video communication. This decision could be influenced by the psychosocial nature of chronic pain treatment [81]. In the treatment of chronic pain especially, patient-therapist communication plays an important role, as pain should be defined as a subjective phenomenon in the discussion and both empathy and emotional support are considered necessary [82]. Although touch is not essential to convey empathy and create a therapeutic bond per se [83], a qualitative study in patients with chronic pain has shown that some patients associated remote patient-therapist counseling with a loss of personal attention [84]. Therefore, it seems that in order to allow patients to recover faster and increase their satisfaction and use of telerehabilitation systems, the two rules of patient-therapist communication and empathy and emotional support should always be considered in these systems.

- ***Reliability of telerehabilitation systems***

According to our other findings, some of the included studies proved the reliability of telerehabilitation in the treatment of patients. By conducting a series of clinical trials on three patients with upper limb disability, Song et al. [32] demonstrated the efficiency and good reliability of telerehabilitation techniques in movement therapy at home or in nursing homes. movement Also, this technology is able to improve the efficiency of rehabilitation training and solve the problems of lack of therapists [32]. Furthermore, Sampson et al. [24] assessed the BUiLT + VR system on hemiparesis patients with upper limb disability and discovered that treatment with this system was reliable and could be safely prescribed. The system can also measure isometric power, FMA-UE, and IMI through its positive trend. A prerequisite for reliability is that we evaluate a system to ensure that it performs its intended function over a period of runtime without any failure [85]. Some studies have shown that telerehabilitation-based intervention is somewhat related to concurrent validity and reliability of the outcome standards. So, evaluating and making the concurrent validity and reliability of outcome standards via telerehabilitation prior to adoption in the scheduled clinical practice has been considered imperative [86].On the other hand, to maximize reliability, we must minimize the faults of the systems. Although the definition of failure is different for different systems and different states, a failure is always a part of the system that exists and can be eliminated by correcting the wrong part of the system [87]. Reliability is defined as failure-free operation over time. This definition in health care is linked to several of the IOM's goals for the healthcare system, including "effectiveness (where the failure can result from not applying evidence)," "timeliness (where the failure results from not taking action in the required time")," and "patient-centeredness (where failure results from not complying with patients' values and preferences)" [88]. Therefore, before a rehabilitation system is provided to patients, its reliability must be ensured from different dimensions, leading problems must be prevented, and various reasons or factors that may affect reliability must be considered at all times. The major factors that affect reliability are improper maintenance or installation, operating statuses including the working environment, the operators' effects, the operative duty referring to the ranges of operating stress imposed, the education of the staff involved in use and maintenance, and a lack of adequate supervision [89].

- ***Improved quality of life***

Other findings of our study showed that telerehabilitation could improve the quality of life of patients with upper limb. These findings approve the results of two single-center studies [90, 91], which showed that telerehabilitation significantly improved overall quality of life indices. The results of the study by Cikajlo et al. [35] indicated that telerehabilitation both improved UL function in people with Parkinson's disease and improved the quality of life of these patients. Some studies have also shown that short-term improvements in motor function (BBT, UPDRS III) and daily activities lead to improved cognitive function and quality of life in participants with PD without changes in diet, medication, or lifestyle. After analyzing the data obtained from the Stroke Impact Scale (SIS) questionnaire and patient interviews in the study of Pickett et al. [17], it is proved that the use of the Tele-CIMT rehabilitation system reduced patients’ travel to rehabilitation centers, reduced patients' feelings of isolation and improved their quality of life. It is important for us to know that quality of life is not just a dimension that can be easily improved and enhanced. Quality of life, defined as the quality of life affected by the disease, is a multidimensional measure that includes physical, social, and emotional health [92]. Therefore, these three dimensions should be considered when using telerehabilitation. Also, Some studies have shown that upper limb function, mental health, and participation in leisure activities are key variables that affect and enhance the quality of life [92]. Therefore, if we use special interventions such as game-based virtual reality and robot-assisted therapy along with telerehabilitation, the upper limb function, mental health, and participation in leisure activities can be improved [92, 93]. Game-based virtual reality and robot-assisted therapy technologies can increase patients' motivation and desire to perform therapeutic exercises [32, 94, 95], perform repetitive and long exercises easily [96], reduce the time required to perform therapeutic exercises [97], increase the patient's independence in performing therapeutic exercises [97–99], and increase adherence to rehabilitation exercises [100]. Finally, these technologies can help improve the quality of life of patients with upper extremity disabilities. Levy et al. [101] evaluated functional outcomes, health-related quality of life (HRQoL), and satisfaction in a group of veterans through a home video-based telerehabilitation program. Assessments showed significant improvements in functional independence, Montreal Cognitive Assessment (MOCA), two-minute walk test, Veterans RAND 12-ItemHealth Survey (VR-12), and HRQoL. In addition to providing rehabilitation exercises through the telerehabilitation system, the therapist should be able to provide teleconsultation services to patients to improve the mental health status of the patient. Larson et al. [102] also believe that increasing patients' access to symptom management and emotional support services may lead to patients taking a more active role in their healthcare and could improve patient outcomes, including overall quality of life. Larson et al [102] Moreover, the patient’s sensation of control may be strengthened if the patient is invited to participate in setting realistic goals for outcomes in rehabilitation. This suggests that working with patients to set goals for recovery and self-care may improve adherence to these goals over time and improve quality of life. Failure to adhere to lifestyle changes and desired goals at the end of a rehabilitation program is a well-known problem in rehabilitation [103]. Another important issue worth pointing out is that the cultural and religious factors could significantly affect the level of quality of life, so both factors must be considered in telerehabilitation systems to improve the quality of life [104].

“Increasing the total exercise time” was the only negative outcome identified in our study [16]. This outcome is discussed below.

- ***Increasing the total exercise time***

A study by Kuttuva et al. [16] showed that the use of telerehabilitation increased total exercise time (up 28%). In this study, a 56-year-old male with right hemi-paresis underwent telerehabilitation for 3 days/week for 12 sessions. It seems that if this study had been performed with larger sample size and longer intervention time, another result might have been obtained. Tabak et al. [105] argued that if sample size and intervention time were not sufficient in a telerehabilitation study, the main effect of the intervention might not be properly apparent. For example, they believed that four weeks of intervention and 30 participants were not enough to estimate the duration of exercise, establish changes in activity behavior, investigate the treatment effects, or detect exacerbations. Therefore, future studies are proposed with a larger sample size and long intervention time, which analyze treatment effects and compliance on both the short- and long-term. The effects of the telerehabilitation systems maybe be various in the long-term follow-up [106]. As a result, evidence of health benefits from long-term patient follow-up is significant enough to be included in the total exercise increase time with telerehabilitation.

## Limitations of the study

There were a few limitations in this review. In this study, only studies in English were reviewed; if a study was published in a language other than English, we did not review it. However, the present study is the first scoping review to examine the outcomes of telerehabilitation for upper limb disabilities; therefore, the results of this study can serve as a basis for further studies. Also, to find related studies, we searched three scientific databases, Scopus, PubMed, and Web of Science. As a result, future studies should be conducted on a larger number of databases in order to obtain more comprehensive results. Furthermore, we did not perform a critical appraisal of individual sources of evidence in this study; this limitation should be considered in future studies.

## Conclusion

According to this review, telerehabilitation technology is a valuable treatment option in the recovery process for upper limb disabilities, and it has the potential to become a quality rehabilitation services delivery model. Furthermore, this technology is a viable alternative rehabilitation approach for upper limb disabilities, as well as a potentially effective tool for increasing positive behavioral change in upper limb disabilities toward a more physically active lifestyle. It should also be noted that the different aspects of telerehabilitation technology identified in this study can be used to design and implement telerehabilitation systems for other disabilities.

## Supplementary Information

**Additional file 1: Appendix A.** The keywords and searchstrategies used in the PubMed and Web of Science databases are listed in Table1. Table1. Keywords and searchstrategy. **Appendix B. **Overview of the functionalities and facilitiesof telerehabilitation systems presented in the studies. **Appendix C. **Evaluationsand a summary of reported outcomes in the included studies.

## Data Availability

The datasets used and/or analyzed during the current study are available from the corresponding author on reasonable request.

## Abbreviations

UL: Upper limb
CIMT: Clinic-based Constraint-Induced Movement Therapy
TBI: Traumatic Brain Injury
SCI: Spinal Cord Injury
CVA: Cerebrovascular Accident
BUiLT: Bilateral Upper Limb Coach
SSc: Systemic Sclerosis
ABI: Acquired Brain Injury
PD: Parkinson’s Disease
VR: Virtual Rehabilitation
MCITE: Modified Constraint-Induced Therapy Extension
HoVRS: Home based Virtual Rehabilitation System
FMA-UE: Fugl-Meyer Upper Extremity score
WMFT: Wolf Motor Function
MAL: Motor Activity Log
ROM: Range Of Motion
BBT: Box and Block Test
PDQ-39: Parkinson’s Disease Questionnaire
WUSPI: Wheelchair User’s Shoulder Pain Index
IMI: Intrinsic Motivation Inventory
SRQ: Shoulder Rating Questionnaire
UPDRS: Unified Parkinson’s Disease Rating Scale
SIS: Stroke Impact Scale
DASH: Disabilities of Arm, Shoulder, and Hand
QOM: Point Quality of Movement
DXA: Dual energy X-ray Absorptiometry
pQCT: Peripheral quantitative Computed Tomography
CT: Computed Tomography
SF-MPQ: Short-Form McGill Pain Questionnaire
SUS: System Usability Scale
FIM: Functional Independence Measure
ReWiiRe: Wii Rehabilitation
HEPs: Home Exercise Programs
HoVRS: Virtual Rehabilitation System
HAMIS: Hand Mobility in Scleroderma
FIHOA: Functional Index of Hand Osteoarthritis
JTHFT: Jebsen-Taylor Hand Function Test
AAUT: Actual Amount of Use Test
9HPT: Nine-Hole Peg Hole Test
SIS: Stroke Impact Scale
HRQoL: Health Related Quality of Life
MOCA: Montreal Cognitive Assessment
