# Supplementary material for: Telerehabilitation for upper limb disabilities: a scoping review on functions, outcomes, and evaluation methods
Source: Arch Public Health. 2022 Aug 23;80:196. doi: 10.1186/s13690-022-00952-w (PMC9400266; doi:10.1186/s13690-022-00952-w)
Supplement: Supplementary file 1 — Additional file 1: Appendix A. The keywords and searchstrategies used in the PubMed and Web of Science databases are listed in Table1. Table1. Keywords and searchstrategy. Appendix B. Overview of the functionalities and facilitiesof telerehabilitation systems presented in the studies. Appendix C. Evaluationsand a summary of reported outcomes in the included studies. [file 13690_2022_952_MOESM1_ESM.docx]

**Appendix A**

The keywords and search strategies used in the PubMed and Web of Science databases are listed in Table 1.

**Table1**. Keywords and search strategy

| Keyword categories | Keywords |
| --- | --- |
| 1 | (telerehabilitation OR remote rehabilitation OR virtual rehabilitation OR telemedicine OR tele-medicine OR tele-care OR tele-health OR telehealth OR telecare OR remote care OR remote medicine OR teleconsultation OR tele-consultation OR remote consultation) |
| 2 | (upper extremity disability OR upper limb disability) |
| Search strategy | [( 1) AND (2) ] |

The following search strategy was used in the Scopus database.

((“telerehabilitation” OR “remote rehabilitation” OR “virtual rehabilitation” OR “telemedicine” OR “tele-medicine” OR “tele-care” OR “tele-health” OR “telehealth” OR “telecare” OR “remote care” OR “remote medicine” OR “teleconsultation” OR “tele-consultation” OR “remote consultation”) AND (“upper extremity disability” OR “upper limb disability”))

**Appendix B.** Overview of the functionalities and facilities of telerehabilitation systems presented in the studies

| **Ref** | **Functions and services of provided with telerehabilitation technologies** | **Hardware used in providing telerehabilitation services** | **Type of service** | | **Technologies used to provide telerehabilitation services** | | | **Robot type or smart wearable** | **Hardware used to design robots or smart wearable’s** |
| --- | --- | --- | --- | --- | --- | --- | --- | --- | --- |
|  |  |  | **Asynchronous** | **Synchronous** |  |  |  |  |  |
|  |  |  |  |  | **Virtual reality** | **Robots** | **Smart wearable’s** |  |  |
| (10) | Telephone communication between patients and therapists, the ability to hold video conferences, the possibility of recording and sending rehabilitation exercises by patients to the therapist, observing the motor function and movement of the patient's arm on a computer monitor | Telephone, PC workstations, one located at the patient’s domicile and the second at the rehabilitation hospital., two ISDN (128 kbits/sec) modems, camera, 3D motion tracking system (Polhemus 3Space Fastrack, Vermont and wearable device |  | √ |  |  | √ | Glove | D motion-tracking receivers |
| (11) | Patient and physician registration in the system, setting profiles, providing rehabilitation exercises to patients, performing rehabilitation exercises by the patient and sending to the doctor, monitoring the progress and improvement of patients by the physician, receiving feedback on improving the condition from the physician, prescribing exercises and new rehabilitation by the physician according to the patient's health status, providing educational information to patients, providing motivational and encouraging sentences to patients, providing progress charts to the patient and physician | Computer, traditional mechanical mice, wireless gyroscopic mice, force feedback mice, and force feedback joysticks | √ |  |  |  |  |  |  |
| (12) | Designing therapeutic exercises by the physician, changing and controlling rehabilitation impedances based on the patient's performance by the physician, physician monitoring the patient's performance and the recorded therapeutic exercises, response to instant messaging (IM), computer-based telephone conference at UniTherapy, Communicating between two patients via audio, video and text | UniTherapy supports force-feedback joysticks, force-feedback driving wheels, various pointing devices (e.g. mouse, trackball) and keyboard | √ | √ | √ |  |  |  |  |
| (13) | Measurement and recording of range of motion by trackers, sending data and treatment exercises to the physician, monitoring and evaluation of therapeutic exercises by the physician | Web camera, speaker, microphone, personal computer | √ | √ | √ |  |  |  |  |
| (14) | Telephone communication, video conferencing between patient and therapist, physician online monitoring of patient rehabilitation exercises | Polycom Viewstation, Sony 27-inch monitor for viewing the patient at the remote treatment site and Sony VCR (Hi Fi, 4 Head) for taping therapist- trainee interactions (hub site only) |  | √ |  |  |  |  |  |
| (15) | Saving and sending rehabilitation exercises, comparing current rehabilitation exercises with previous exercises, registering patient and therapist, providing new exercises to patients by the therapist according to their progress | Personal computer-based cameras and microphone | √ |  |  |  |  |  |  |
| (16) | Recording rehabilitation exercises, measuring movement recovery, providing progress charts to the patient and physician, the possibility of monitoring rehabilitation exercises by the therapist and prescribing new therapeutic exercises | Camera and a personal computer | √ |  | √ |  |  |  |  |
| (17) | Prescribing therapeutic exercises by the physician, providing progress charts to the patient and the physician, the therapist supervising the rehabilitation exercises performed by the patient, therapeutic education to the patient | Computer, a web cam, and the gripper | √ | √ | √ |  |  |  |  |
| (18) | Providing rehabilitation training to the patient, recording exercises by the patient, sending exercises to the therapist, prescribing new rehabilitation exercises for the patient by the therapist | DSL (digital subscriber line) modem/router, computer and wearable device | √ | √ | √ |  | √ | Glove | Videogame console |
| (19) | Providing custom rehabilitation games to each user, recording patient rehabilitation activities, monitoring and controlling activities by the therapist | A 5DT 5 Ultra Glove (Fifth Dimension Technologies, Persequor Park, South Africa), a 26-inch high-definition TV, a keyboard, and a PlayStation3 game console, modem/router and wearable device | √ | √ | √ |  | √ | Glove | Not mentioned |
| (20) | Providing rehabilitation exercises to patients by the therapist, communication with the therapist and the possibility of reviewing rehabilitation exercises by physiotherapists | PC (or laptop) with webcam |  | √ | √ |  |  |  |  |
| (21) | Performing shoulder flexion, extension and abduction rehabilitation exercises in real time with the help of Bilateral Upper Limb Trainer games and rehabilitation device and remote monitoring of patients by the therapist | PC (or laptop) with webcam |  | √ | √ |  |  |  |  |
| (4) | Providing educational-therapeutic services through video conferencing to the patient | Laptop, 3D motion monitor |  | √ | √ |  |  |  |  |
| (22) | Storing clinical data and rehabilitation exercises, guiding the patient in performing the exercises, providing visual and audio feedback (alarm), providing adequate statistics (e.g. maximum, minimum, average time, speed, etc.) to the patient and the physician, determining the patient's adherence to the treatment protocol | Tool for hand grip and pich, tool for hand abduction, charger, antenna, tool for finger tapping, tool for dynamic rotation, tool for isometric rotation, control display and thermometer | √ |  |  |  |  |  |  |
| (23) | Recording rehabilitation exercises and sending to the therapist, retrieving rehabilitation exercises by physician and prescribing medication and new rehabilitation exercises | Computer or laptop, microphone and web cam | √ | √ |  |  |  |  |  |
| (24) | Providing various rehabilitation services to the patient through video conferencing | Camera, microphone, a web cam |  | √ |  |  |  |  |  |
| (25) | Video calls | Kinect, polarized display (Mitsubishi RDT234WX-3D), polarized glasses, web camera and wearable device |  | √ | √ |  | √ | sensory feedback system | Kinec, vibrator devises, A polarized display |
| (26) | Prescribing therapeutic exercises by the therapist, the therapist supervising the rehabilitation exercises performed by the patient, conducting video conferences between the patient and the physician | Personal computer, web camera |  | √ |  |  |  |  |  |
| (27) | Video conferencing, user management capabilities and patient status (online, offline, previous sessions, scheduled sessions), secure video, audio and data transfer over the Internet | Touch monitor, a mini-PC (Intel NUC), a pan-tilt-zoom (PTZ) camera with embedded h264 video codec, a microphone array and a speaker |  | √ |  |  |  |  |  |
| (28) | Prescribing a set of new rehabilitation exercises by the therapist, selecting the exercises by the patient from the library, displaying 3D arm images, recording the rehabilitation exercises performed by the patient and sending to the therapist, providing encouragement messages to the patient, retrieving the exercises sent by patients by the therapist, monitoring the patient's progress by the therapist, determining the range of motion, speed, duration and number of repetitions of each rehabilitation exercise by the therapist, giving the patient a score by the therapist based on the exercises performed and providing medical feedback to the patient by the physician, selection of games by the patient, according to the degree of disability and their needs | Wii Remote and personal computer | √ |  | √ |  |  |  |  |
| (29) | Patient and physician registration in the system, physician video communication with patients, providing necessary training to patients to control and manage the robot, recording therapeutic exercises performed by patients, accessing to the history of rehabilitation exercises and data obtained from the robot sensors, providing progress charts on improving performance to the patient and therapist, retrieving rehabilitation exercises stored by the patient and physician, saving audio signals | Web-based server computer, client computer, a webcam and a microphone and three telerehabilitation robots |  | √ | √ | √ |  |  | Position sensor, DC torque motor, encoder, force / torque sensor, robot arm, support handle |
| (30) | Recording rehabilitation exercises by patients and sending them to physiotherapists, providing home rehabilitation exercises to patients and providing motivational messages to patients | Smart phone, tablet or computer, webcam | √ | √ | √ |  |  |  |  |
| (31) | Educating patients, giving scores or encouragement to patients if they do therapeutic exercises correctly, determining the level of each rehabilitation activity by the therapist for patients according to their abilities and functions | Personal computer, large television monitor and Microsoft’s Kinect 3D sensor | √ |  | √ |  |  |  |  |
| (32) | Recording exercise therapy services and reviewing these services by the therapist, prescribing new exercises after improvements in the upper extremities, retrieving data stored by the therapist for further review, adding therapists and new patients to the system, setting the game by therapists and updating data stored in the database | Three-dimensional infrared camera, Kinect V2; Kinect V2 SDK, LCD TV (HDMI input) and PC | √ |  | √ |  |  |  |  |
| (33) | Providing different rehabilitation exercises to the patient to pull the wrist, hold and release the hand, determining the type and content of games by the patient, determining the difficulty level of rehabilitation exercises by the therapist based on the patient's health status, monitoring the patient's performance by the therapist | Microsoft Kinect Sensor, Camera and computer | √ | √ | √ |  |  |  |  |
| (34) | Face-to-Face video communication between patient and therapist and remote measurement of patient performance by therapists | A computer with an integrated microphone and camera |  | √ |  |  |  |  |  |
| (35) | Teaching rehabilitation exercises to the user through games, monitoring the patient's rehabilitation exercises by the therapist, showing the number of correct movements or objects to the patient, adding encouragement or applause sounds to the exercises to enjoy more patients, alerting patients when needed to perform compensatory movements | Kinect and computer | √ | √ | √ |  |  |  |  |
| (36) | Registration of patients and physicians in the system, prescribing therapeutic exercises to the patient by the physician, sending the exercises performed to the therapist by the patient, the possibility of choosing the game or rehabilitation exercises by the patient, assessing the patient's disability by the physician, monitoring patients' progress, adding a new doctor to the system, deleting and updating rehabilitation exercises, adding or removing a patient from the system, updating patient, tracking patients, setting game, selecting the injury date by the patient, determining the injury level and selecting the game | Personal computer, camera and wearable device | √ | √ | √ |  | √ | Glove | Microcontroller, Accelerometer and  Gyroscope Sensor, Flex sensor , heart rate sensor |
| (37) | Designing custom rehabilitation exercises by therapists, face-to-face interaction between patient and physician, online recording of rehabilitation exercises performed by the patient, reporting of rehabilitation exercises performed, providing progress charts to patients | Infrared camera, computer | √ | √ | √ |  |  |  |  |

**Appendix C.** Evaluations and a summary of reported outcomes in the included studies

| **Ref** | **Duration of use of the systems** | **Evaluation Methods/tools** | **The number of participants in the evaluation process** | **Outcomes of using telerehabilitation systems** |
| --- | --- | --- | --- | --- |
|  |  |  |  |  |
| (10) | 4 weeks (daily 1-hour of VR tele-therapy, 5 days per week) | -Assessment of the arm motor performance and the activities of daily living using the Fugl-Meyer and Functional Independence Measure scale, together with the determination of the velocity and duration of 10 representative reaching movements. | Five patients suffering from mild/intermediate arm motor impairments | - Significant improvement in the mean score of Fugl-Meyer ((Fugl – Meyer UE mean score mean duration and mean velocity of reaching movements by 10.5, 20.2% and 29.8%, respectively)) - Significant improvement in the duration and speed of exercises - A slight improvement in the Functional Independence Measure (FIM) score - Improved arm motor movements - High patient satisfaction with the designed system |
| (11) | 12 weeks | Feasibility of using the system (to direct a therapy program, mechanically assist in movement, and track improvements in movement ability) | 1 subject with chronic stroke | - Feasibility of the system to guide the treatment program, mechanical assistance in improving movement and continuous follow-up of treatment - Providing cost-effective and affordable rehabilitation services |
| (12) | - | -Evaluation of the subject’s neuromotor performance (the discrete cross tracking task to track a 5 point cross three times separately by both unaffected side and affected side) | 16 subjects (8 subjects with stroke-induced disability and 8 normal( | - Improved musculoskeletal function in both groups - Using UniTherapy as an adult clinical research tool to integrate different rehabilitation exercises in a single context |
| (13) | 5 weeks | Fugl-Meyer15 test before and after treatment | 1 chronic stroke subject | - Improvements in arm motor control and shoulder range of motion according to the Fugl-Meyer scale - Improving the duration for each rehabilitation exercise - Increased patient motivation to continue treatment - Increased total exercise time by 28% - Increased total wrist translation motion per session by up to 90% - Increasing the speed of performing sports movements - Increased patients' motivation |
| (14) | 3 months | - Functional assessment with the WMFT, the MAL, the Actual Amount of Use Test (AAUT), and the Box and Block Test (BBT)  - Measuring of quality of life with the Stroke Impact Scale (SIS)  - Grip strength assessment using a standard grip dynamometer  - Evaluation of functional skills of two stroke survivors completed through clinic-based CIMT and, subsequently, a home-based CIMT trial (tele-CIMT) incorporating telecommunications technology | Two subjects with stroke | - Improving the intact cognitive functioning with unit exception of shortages in the ability to discriminate emotions with neuropsychological testing - The absence of aphasia or movement-speech disorder - Improved WMFT scores - Providing more complex patterns for two AAUT actions - Increased AAUT score for rehabilitation offered through clinic-based CIMT and tele-CIMT - Improving BBT scores for rehabilitation offered in the clinic – CIMT - Improving motor skills in both face-to face intervention and tele-CIMT methods - Partial confirmation of the effectiveness of tele-CIMT as an alternative treatment - Increased adherence to therapeutic exercises |
| (15) | 30-minute therapy sessions, 3 times a week for 10 weeks | -The Motor Activity Log (MAL) and WMFT Test  -Structured interview for satisfaction. | Four people with stroke | - More use of the arm (+2.7, +2.06, +1.7, +2,83, respectively) after intervention - Improving the quality of movements (+2.1, +2.1, +2.03, +1.9, respectively) - Increasing the satisfaction of the participants - Increased adherence to treatment - Good feasibility of telerehabilitation system |
| (16) | 6 months (15 therapy sessions, 3 times per week) | -Comparative clinical evaluation | 22 stroke patients (a control group with 11 patients and a study group with 11 patients) | - Improved arm mobility and function - Improvement of Motricity index in the control group from 42.1% to 52.6% and in the study group from 32.33% to 52.91% - Improvement of Fugl-Meyer scale from 18 to 26.3 in the control group and from 13.41 to 31.91 points in the study group - Important perceptual experience in terms of effort and utility in the study group - Similarity of the traditional method of rehabilitation and Gesture Therapy system in reducing pressure and pain in both groups - Enjoy more treatment and more interest in using the Gesture Therapy system - Cost-effectiveness of telerehabilitation system compared to traditional rehabilitation methods - Increasing the motivation of patients to perform therapeutic exercises in the group using Gesture |
| (17) | 7 weeks) 3 sessions per week about 60  minutes each) | - Functional evaluation: with the Fugl–Meyer scale and the Motricity Index and an Intrinsic Motivation Survey  . | 42 subjects with stroke(control group with 22 patients, and a study group with 20 patients) | - Improved motor and functional arm in both groups (according to the Wilcoxon statistical test with p <0.5) - Significant improvement in Motricity index (from 18 to 26.3 in the control group, and from 19.34 to 31.36 in the study group) - Significant improvement in the Fugl – Meyer intervention group compared to the study group (from 18 to 26.3 in the control group, and from 19.34 points to 31.36 points in the study group) - Greater improvement in the study group for both scales; with a difference of 30.00% vs. 7.4% for the Motricity Index, and 12.02 points vs. 8.3 for the Fugl Meyer scale - More pleasure and enthusiasm of the study group from the Gesture therapy system - Patients' efforts to make greater use of the system to improve performance - Using the gesture therapy system at home without the need for a therapist - Cost effectiveness of Gesture therapy system - Similar effect of Gesture rehabilitation system and traditional rehabilitation in terms of standard clinical scales - Increasing patients' motivation and their greater dependence on treatment - Enjoy more rehabilitation processes and be more interested in using the Gesture Therapy system - Reduced medical costs |
| (18) | 3 months (30 minutes a day, 5 days a week) | -Standardized occupational therapy assessments, remote assessment of finger range of motion (ROM) based on sensor glove readings, assessment of plegic forearm bone health with dual-energy x-ray absorptiometry (DXA) and peripheral quantitative computed tomography (pQCT), and functional magnetic resonance imaging (fMRI) of hand grip task. | 3 subjects with severe hemiplegic cerebral palsy | - Improving hand function and arm bone health (as measured by DXA and pQCT) - Confirmation of improved paralyzed hand function through occupational therapy testing - Advances in radial bone mineral content and in the plegic limb in two patients - Increased spatial degree of activation at after care relative to baseline in brain motor circuitry by fMRI during grip task contrasting the plegic and nonplegic hand - The good performance of the remote-rehabilitation in developing and amended hand performance and arm osseous tissue health - Increased use of the system by patients - Advance on grip testing and the Jebsen test, such as a clinically meaningful developed power to lift lightly and heavily targets - Activating clinical exam motor cortex and cerebellum bilaterally |
| (19) | 14 months | Jebsen-Taylor Hand Function Test (JTHFT), Forearm bone health assessment using dual-energy x-ray absorptiometry and peripheral quantitative computed tomography | 1 subject hemiplegic cerebral palsy and epilepsy | - Improved plegic hand function by measuring the Jamar dynamometer index from 4 pounds to 9 - Improving patients' performance in performing basic functions - Performance improvement in performing activities related to imitative page turning, picking up light things, simulated feeding, and lifting big or small things - Improved bone health in the upper extremities - Reduction of paretic upper extremity to nonparetic discrepancy in bone mineral content in distal and supra distal radius - The effectiveness of the system to improve upper limb function |
| (20) |  | -Muscles functional status assessment with EMG sensors(By doing rehabilitation exercises and then evaluating with a questionnaire) | 10 healthy subjects | - Increased range of motion in the upper extremities - Increasing the level of interest and pleasure of patients to perform therapeutic exercises - Increased patients' motivation to perform rehabilitation exercises - Solve the challenge in deficit of therapists |
| (21) | 6 weeks(45 min, 4 days/week( | -Functional status assessment( Use of the Fugl-Meyer Upper Extremity score (FMA-UE) as the primary outcome measure)  isometric strength and the Intrinsic Motivation Inventory (IMI) assessment with questionnaire | Five post-stroke participants with UL hemiparesis( one sub-acute and four chronic) | - Improved musculoskeletal function of different parts of the UL - Increasd FMA-UE scores from 1 to 5 and overall strength in the shoulders and elbows through BUiLT + VR treatment - Increased patients' motivation to perform therapeutic exercises - Increased isometric strength in the shoulders and elbows - No fatigue - Increased motivation of patients to perform therapeutic exercises - Increased the use of the system to perform rehabilitation exercises - Reliability of BUiLT + VR system - Cost effectiveness of the system |
| (4) | 6 weeks (5 days/week, 60 min/day) | -Lab-based clinical and kinematic assessments( laboratory-based pre-, post- and one-month follow-up testing consisting of the Streamlined WMFT Test, kinematic assessments of unilateral reaching movements from a waist to shoulder height target using an electro-magnetic 3D motion monitor (Ascension Technology Corporation, Burlington, VT), computerized cognitive assessments using CogState software (New Haven, CT) and position sense) | Seven adults with chronic stroke | - Improved tactile discrimination function in the less impressed hand (degrees of freedom (df) = 6, z = 2.207, p = 0.03) - Trended towards improvement in the more impressed hand (df = 6, z = 1.859, p = 0.06) - Improving the movements of the injured arm after the intervention - Improvements in sensory tasks - Reduced number of errors in the Groton maze learning test - Improved cognitive performance in five participants - Approved remote telerehabilitation program as a convenient method - Possibility of profound changes in care methods related to the treatment stages of chronic stroke |
| (22) | 3-month | -Hand function measured by Dreiser's index (Functional Index for Hand OA, FIHOA), Health Assessment Questionnaire (HAQ), and the Hand Mobility in Scleroderma (HAMIS) test (only for SSc). | Ten patients with systemic sclerosis (SSc) and 10 with rheumatoid arthritis (RA) | - Advances in sensational tasks (degrees of freedom (df) = 6, z = 2.207, p = 0.03) - Improved FIHOA in both arms - Improvement of HAQ and HAMIS test results only in the tested arm - Improving the condition of the arm tested in patients with RA based on FIHOA and HAQ - Confirmation of cognitive function improvement through Groton Maze Learning Test (df = 5, z = 1.68, p = 0.09) - Lack of statistically significant differences in outcome measures between treatment methods - Increased adherence to treatment - Possibility of intervention and remote control of therapeutic exercises by the therapist |
| (23) | 8 weeks ( 45 mins, 5 days per week) | -Limb position sense assessment with using three conditions: ipsilateral remembered (same arm used for reference and matching targets), contralateral concurrent (reference arm moved and held at target position while opposite arm matched reference position), and contralateral remembered (reference arm moved to target position and then returned to start position before opposite arm matching position). | 12 subjects with cerebral palsy | - Fewer absolute mistake crosswise matched tasks and no modification in constant error - Reduction of absolute error of participants after training and use of the system and no change in constant error - Improving sensory-perceptual function between the arms (F_1,541.87_ = 8.875, P < 0.01)) - Under the influence of absolute error for the cognitive task performed and the arm used (F_2,532.94_ = 2.999, P = 0.05) - No significant difference in performance between the arms (P> 0.59) - Confirming the role of the system as potential for reinforced proprioceptive function after education in movement and unrelated sensory tasks |
| (24) | 8 weeks (30 to 45 minutes per day) | -Pain (Short-Form McGill Pain Questionnaire [SF-MPQ]), disabilities including shoulder range of motion (flexion, extension, internal rotation, external rotation, abduction), and upper limb function (DASH) Index)  - Participant satisfaction (assessment with the Health care satisfaction questionnaire) | 17 with proximal humerus fractures | - Improving the motor-functional condition of the upper limb - Reducing and relieving pain after intervention - Improving the consequences of shoulder ROM for various dimensions of flexion, extension, internal rotation, external rotation, abduction - High patient satisfaction with the services provided (82.7%) - Increased patients' motivation to use remote rehabilitation service |
| (25) | 20 days | Effectiveness of the system (by analysis of the recovery with motor paralysis)) | 10(Five healthy persons and five people with motor paralysis) | - Increased flexion of the shoulder joint - Increased flexion of the elbow joint - No delay in data transfer - Reduced the time required to perform rehabilitation and balance exercises for patients - No side effects - Effective and safe telerehabilitation system |
| (26) | 12-weeks (3 sets of 30 repetitions, 3 times weekly) | -Functional status assessment )Wheelchair User’s Shoulder Pain Index (WUSPI), Disabilities of Arm, Shoulder, and Hand (DASH) Index, and Shoulder Rating Questionnaire (SRQ)( | (N = 16, 13 men/3 women; 15 who had a spinal cord injury, 1 post polio | - Performance improvement (WUSPI (χ22 = 5.10, P = .014); DASH Index (χ22 = 5.41, P = .012); and SRQ (χ22 = 23.71, P = <.001)) - Reduced pain - Increasing the strength of isometric indices of serratus anterior and scapular retractors - Improved muscle nerve impulse by the lower trapezius during a fatigue task - Promising use of high-dosage scapular stabilizer and rotator cuff increasing platform in remote rehabilitation and monitoring of shoulder pain treatment - Increased adherence to treatment (> 75%) - No significant difference in isometric strength for the lower trapezius muscle, glenohumeral rotators, and abductors - Providing a stabilizer and rotator cuff strengthening platform as a potential to prevent shoulder pain |
| (27) | 8 weeks(30 to 45-min sessions) | - Upper extremity function (Constant Shoulder Score and Disability of the Arm, DASH Index  - Range of motion (conventional goniometer);  -User satisfaction (Health Care Satisfaction questionnaire); and cost of services to the public healthcare system. | 52 participants (26 per group) | - Access to fast, cheaper and satisfactory rehabilitation services - Demonstrate the good performance of telerehabilitation system compared to face-to-face visit |
| (28) | 2 weeks | -Accuracy of the system(the system was compared with a well-established professional and accurate system, namely the ViconMoCap Studio.3) | Three stroke survivors | - Recovering the motor-functional capablenesses of people - A large recovering  in grade on the NHPT (from 29 to 7.8 s per peg) - Recovering  in disabilities in FMA (an increment of 8 points from 39 to 47 on a 66 point measure) - Recovering in grade for functional spontaneous use of the arm (MAL grade from 7 to 10 on a 70 point measure) - Normalized accuracy range between 5.5% and 10.78% for Root-Mean-Square Deviation (NRMSD) - Increased finger and wrist flexibility - Calibrating activities individually - Acceptability of using Low-cost and off-the-shelf game sensors in rehabilitation processes - No side effects - Increased speed and range of motion - Increased patients' motivation to continue treatment and adhere to it - Increased use of rehabilitation exercises |
| (29) | 4 weeks | Clinic experiments (measuring the movement of the patient arm and the interaction force between the patient arm and the robot) | Three patients with stroke | - Improve the function of different parts of the upper limbs - Improve muscle strength and movement coordination - Good system reliability and improve efficiency of the rehabilitation training - Solve the problem of lack of therapist |
| (30) | 4 weeks | -Functional performance, disability, patient satisfaction, perceptions of treatment effectiveness, and different aspects of adherence | Eighty participants )intervention (n=40) or control (n=40) groups( | - Significant difference between control group and intervention in performance improvement, self-reported exercise and adherence to treatment (in favor of the intervention group with a value of 1.3 / 11 and 95% CI 0.2 to 2.3) - The average between-group deviation for performance was 0.9/11 points on the Patient-Specific Performance Scale (privileging the intervention group) - No significant difference in patient satisfaction between the two groups of health services (95% CI -0.3 to 1.3) - Increased adherence to treatment (at 4 weeks was 1.3 / 11 points (95% CI 0.2 to 2.3)) - Patient satisfaction with supportive medical services (95% CI -0.5 to 1.5) - Lack of significant differences in other outcomes between intervention and control groups |
| (31) | 30-min biweekly sessions | -Clinical assessments ( measured movements and function of the weaker upper extremity and cognitive abilities)  - Usability testing (System Usability Scale (SUS)) | 82 subjects (46 males; 74 with ABI)  - Usability testing(6 with ABI) | - The effectiveness of the CogniMotion System in providing clinical outcomes - Improved muscle strength and motor coordination - Improved patient satisfaction with the services provided - Significant improvements in FMA score (n=35)) z=-3.1; p=0.002, shoulder flexion (n=42) z= 3.02) - No significant change in the amount or quality of MAL and in TMT-A   Usefulness of the system for rehabilitation (mean ± SD= 89.1 ± 12.1)   - Increased participants' enjoyment from their experience with the service (mean ± SD= 4.1 ± 1.1) - CogniMotion System feasibility for people with ABI |
| (32) | over 3 weeks | -Functional status assessment([Unified Parkinson’s Disease Rating Scale (UPDRS)), Box and Block Test (BBT), Nine-Hole Peg Hole Test (9HPT);], and daily functional tasks evaluation  -Parkinson’s Disease Questionnaire (PDQ-39) to estimate the health status over the last month.) | 28 patients with PD | - Statistically significant clinical outcomes related to Box and Blocks Test (mean: 47 vs. 52, P = 0.002, Cohen's d = 0.40), UPDRS III (average: 27 vs. 29, P = 0.001, d = 0.22), and daily activity Jebsen's test; writing a text (average: 24.0 vs. 20.6, P = 0.003, d = 0.23); and going light targets (average: 4.4 vs. 3.9, P = 0.006, d = 0.46) - Reliability of BUiLT + VR system - Improving the cognitive functions and quality of life of participants with PD without changing their medication regimen and lifestyle - Improved motor function in patients with PD over time - Spend less time doing rehabilitation exercises - Normal BBT data (kurtosis / skewness: 0.51 / 0.1 earlier and .0.32 / 0.59 later participation) and the UPDRS III (kurtosis / skewness −0.32 / −0.35 earlier and −0.11 / 0.09 later participation) |
| (33) | 10 days (5 days per week) | Functionally assessment (the Fugl-Meyer Assessment (FMA) (primary outcome) and other secondary functional outcomes and accelerometers assessment to measure hemiparetic upper limb movements during the therapy) | 23 participants ( sham (n = 11) and real (n = 12) VR ) | - Improved arm and hand function in both sham and VR groups based on BBT, and K-MBI - No significant difference in FMA for both sham (46.8 ± 16.0) and real VR (49.4 ± 49.0) groups - Significant difference in the total number of upper limb activities  in hemiparetic UL during the clinical care between groups  (F2,26 = 4.43; P = .22) - Significant improvements for arm and hand, BBT, and K-MBI in both real and sham VR groups - Increased participation of patients using VR during rehabilitation exercises and their efficient adaptation to the system - Using the system as an adjunctive and useful treatment for rehabilitation of people with stroke |
| (34) | 20 minutes | -Upper limb measures of function (assessed with the Manual Ability Measure 16), dexterity (evaluated using the coin rotation task), motor speed (assessed by the finger tapping test), tremor (evaluated with the Fahn-Tolosa-Marin Tremor Rating Scale), and range of motion (using the Kinovea software( | Twenty-one patients with PD | - Providing a high confidence interval for all assessment criteria related to skill, motor speed and upper extremity vibration - Confidence intervals ranging from 0.63 to 0.93 and from 0.75 to 0.95 in the highest degree and least impacted upper extremity, respectively - For elbow joint flexion, assurance intervals range from 0.49 to 0.89 and 0.67 to 0.93 for the highest degree and least affected - ICC range from 0.63 to 0.93 with the most injuries in the elbow and ICC range from 0.51 to 0.89 in less impacted rotation movements - High reliability in remote evaluation of upper extremities in patients with PD compared to face-to-face evaluation - - Improving the skills of performing rehabilitation exercises - - Improving the degree of flexion of the shoulders, elbows, wrists - - Improving the degree of traction of the shoulders, elbows, wrists - -High reliability of upper limb telerehabilitation system in patients compared to a face to-face evaluation |
| (35) | 3 months | -Functional status assessment with FMA-UE, Wolf Motor Function (WMFT) and Motor Activity Log (MAL) tesets | Thirty-three patients with chronic hemiplegic stroke. (Seventeen patients in the Kinect2Scratch group and 16 patients in the physician-based education group) | - Significant improvements in WMFT-TIME (P=0.004) and MAL (AOU and QOM, P=0.009, 0.003) - Significant improvements in FMA-UE (P=0.001), FMA -PROX (P=0.001) and FMA-DIS (P=0.017) - Significant improvements in FMA-UE and FMA-PROX (P=0.014, 0.005 respectively) and WMFTTIME (P=0.006) - High level of participation in the Kinect2Scratch group compared to the therapist-based training group - Higher number of rehabilitation activities in the Kinect2Scratch group compared to the therapist-based training group - Similar effect of Kinect2Scratch game training method with therapist-based training in improving the UE performance of patients with stroke - Feasible to improve upper limb function - Patients do not need further treatment - No side effects |
| (37) | 3 months(use the system at least 15 min every weekday) | -Feasibility the use of system(15 persons with stroke that participated in pilot testing of HoVRS in their homes) | Fifteen subjects with chronic stroke | - Improved upper limb function capable of safe and continuous use of the system - Feasibility of using HoVRS system as a cost-effective rehabilitation system for rehabilitation processes - Ability to track hand and finger function - Increased arm strength - Increased adherence to rehabilitation treatments - Increased UEFMA mean (SE = 0.69, p <0.001, 95% confidence interval (3.66, 6.71)) - Increased ROM in all three measurements: 15.83% for HOR, 27.50% for WPR, and 37.20% for HRR - Reduction of errors during treatment exercises (15.76% in HOA, 18.70% in WPA and 18.75% in HRA) |
